# Antitumor Properties of Epitope-Specific Engineered Vaccine in Murine Model of Melanoma

**DOI:** 10.3390/md20060392

**Published:** 2022-06-14

**Authors:** Emiliya Stoyanova, Nikolina Mihaylova, Nikola Ralchev, Petya Ganova, Silviya Bradyanova, Iliyan Manoylov, Yuliana Raynova, Krassimira Idakieva, Andrey Tchorbanov

**Affiliations:** 1 Laboratory of Experimental Immunology, Stefan Angeloff Institute of Microbiology, Bulgarian Academy of Sciences, 1113 Sofia, Bulgaria; stoyanova_e@microbio.bas.bg (E.S.); mihaylova_n@microbio.bas.bg (N.M.); nikola_ralchev@microbio.bas.bg (N.R.); pganova@microbio.bas.bg (P.G.); silvybradyanova@microbio.bas.bg (S.B.); iliyanmanoylov@microbio.bas.bg (I.M.); 2 Institute of Organic Chemistry with Centre of Phytochemistry, Bulgarian Academy of Sciences, 1113 Sofia, Bulgaria; yulianaraynova@hotmail.com (Y.R.); Krasimira.Idakieva@orgchm.bas.bg (K.I.)

**Keywords:** C57BL/6 mouse model, epitope-specific vaccines, hemocyanins, anticancer therapy

## Abstract

Finding new effective compounds of natural origin for composing anti-tumor vaccines is one of the main goals of antitumor research. Promising anti-cancer agents are the gastropodan hemocyanins–multimeric copper-containing glycoproteins used so far for therapy of different tumors. The properties of hemocyanins isolated from the marine snail *Rapana thomasiana* (RtH) and the terrestrial snail *Helix aspersa* (HaH) upon their use as carrier-proteins in conjugated vaccines, containing ganglioside mimotope GD3P4 peptide, were studied in the developed murine melanoma model. Murine melanoma cell line B16F10 was used for solid tumor establishment in C57BL/6 mice using various schemes of therapy. Protein engineering, flow cytometry, and cytotoxicity assays were also performed. The administration of the protein-engineered vaccines RtH-GD3P4 or HaH-GD3P4 under the three different regimens of therapy in the B16F10 murine melanoma model suppressed tumor growth, decreased tumor incidence, and prolonged the survival of treated animals. The immunization of experimental mice induced an infiltration of immunocompetent cells into the tumors and generated cytotoxic tumor-specific T cells in the spleen. The treatment also generates significantly higher levels of tumor-infiltrated M1 macrophages, compared to untreated tumor-bearing control mice. This study demonstrated a promising approach for cancer therapy having potential applications for cancer vaccine research.

## 1. Introduction

Cancer has remained the leading cause of death over the last few decades on a worldwide scale, which necessitates the development of various anti-cancer vaccines for the treatment of this malignant disease. Malignant melanoma is a form of skin cancer, and its global frequency is rising at an alarming rate in both men and women [1,2]. The current therapies for cancer are nonspecific and mainly based on chemotherapy and radiotherapy, often disrupting other body functions [3]. Several monoclonal antibodies and different anticancer vaccines which show encouraging results are being studied, but they are far from routine use in clinical procedures as of yet [4,5,6].

Today, the use of medicines derived from natural products has a huge impact on the process of anti-cancer drug development. Marine natural products are known for their huge variety of chemical compounds. The unique structures are often linked to special mechanisms of action, through which they can trigger unexpected biological activities, such as directly affecting tumor processes on the cellular and tissue level by blocking various growth factors. More attractive for modern medicine are molecules with limited adverse reactions affecting tumor progression through enhancing the effective antitumor response by various mechanisms provided by acquired and innate immunity [7,8].

Hemocyanins (Hcs) are large, copper-containing respiratory glycoproteins freely dissolved in the hemolymph of most mollusks and arthropods. When injected into mammals, Hcs enhance the innate and adaptive immune response, based on their xenogenic character and carbohydrate content [9,10,11,12].

Mollusk Hcs have applications both in clinical practice and in the field of immunology as natural non-toxic and non-specific immunomodulators for the treatment of cancer models [12,13,14,15], and as protein-carriers or adjuvants of tumor-associated antigens in the development of cancer vaccines. These giant (molecular size up to 8–9 MDa) oxygen transport glycoproteins elicit a Th1 immune response and are widely used in biomedicine and biotechnology [16,17]. The hemocyanin, isolated from the marine gastropod *Megathura crenulata*, known as Keyhole Limpet Hemocyanin (KLH), is a golden standard for the multiple applications of Hcs.

Furthermore, two other Hcs have been isolated from mollusks found along the Pacific coast of Chile and Peru. The Hcs isolated from the Pacific mollusk *Concholepas concholepas* (CCH) and the one isolated from the Chilean mollusk *Fissurella latimarginata* (FLH) exhibited high immunogenicity and equal or even better anti-tumor properties, compared to KLH in superficial bladder cancer, melanoma, and oral cancer models [12,13,15,18,19].

In our studies, we used two other Hcs, isolated from marine gastropod *Rapana thomasiana* (RtH) and from garden snail *Helix pomatia* (HpH) for therapy of C-26 murine model of colorectal carcinoma based on cross-reactive tumor-associated epitopes. The successful use of Hcs for antitumor therapy depends to a large extent on the immunization schedule, which gives various advantages among the tumor models, as well as depending on the variety of Hcs. Using three different schemes of administration (mild, intensive, and upon priming), both Hcs exhibited a strong anti-cancer effect with changing efficiency relative to the schemes. Immunization of experimental animals with RtH or HpH generated high levels of tumor-specific antibodies and Cytotoxic T lymphocytes (CTL), as well as the secretion of proinflammatory cytokines, leading to inhibition of tumor development and increased survival [20,21]. The same approach of treatment with Hcs (KLH, CCH, or FLH) in a B16F10 mouse model of melanoma resulted in variety of immune responses among the Hcs, probably directed to the different carbohydrate content [15]. We also observed the dominance of immune responses depending on RtH or HpH administration within the same therapeutic schedule in the C-26 murine model of colorectal carcinoma [21].

The overexpression of tumor-associated antigens (TAAs) on the tumor cell surface is a specific feature differing in comparison to the normal tissue. It seems that the Hcs’ carbohydrate overlapping with TAAs is the reason for the diversity in immune mechanisms triggered by Hcs immunizations due to the different pattern recognition by numerous cell receptors. Several gangliosides such as GM2, GD2, and GD3 are identified as TAAs [22]. These glycosphingolipids contain a lipidated reducing end, and their overexpression is found in human melanomas. Based on these findings, several anti-tumor therapeutic vaccines directed to TAAs have been developed and applied in different mouse models [22,23,24].

Palacios et al. used a mimetic peptide of the ganglioside GD2 expressed upon human neuroblastomas, soft tissue osteosarcomas and melanomas for conjugation with two Hcs—CCH and FLH [14]. These constructed molecules have been applied in the B16F10 murine model of melanoma in order to induce antibody dependent cell-mediated cytotoxicity. 

Another possible target is the ganglioside GD3, which is also reported to be a TAA, since it has been found exclusively in melanomas but not in normal melanocytes [25]. The aim of the present research work is the suppression of tumor progression in a mouse melanoma model by chimeric protein vaccines that contain hemocyanin molecules conjugated to a mimotope peptide structurally resembling the tumor-associated carbohydrate epitope GD3. Here we explored two Hcs—one isolated from *Rapana thomasiana* (RtH) and another one from the terrestrial snail *Helix aspersa* (HaH), which are used in various therapeutic schemes in the murine model of melanoma.

A number of experiments and data analyses have been performed such as tumor incidence, tumor growth, survival, phenotyping of tumor cell infiltrates, induction of humoral immune response, and CTL generation, as well as cytokines production and M1/M2 discrimination in tumor-infiltrating macrophages in the animals after in vivo RtH-GD3P4 or HaH-GD3P4 administration. 

## 2. Results

### 2.1. RtH and HaH Purification

Pyrogen-free materials and reagents were used for the isolation and purification of RtH and HaH. The LAL assay of final sterile Hc preparations for the presence of endotoxins showed 4.5 EU/mg protein for RtH and 4.7 EU/mg protein for HaH.

### 2.2. Tumor Development and Survival Analysis

**Figure 1:**
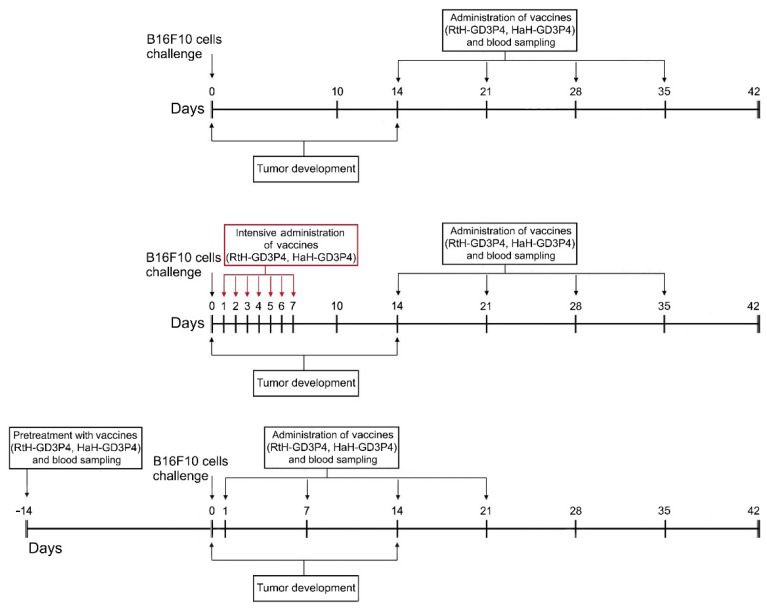
The schedule of the experimental therapeutic design.

The mice of the tested groups were challenged and vaccinated as shown in Figure 1. All mice from the control group were treated with PBS developed palpable solid tumors on day 16 after the B16F10 cells challenge.

**Figure 2:**
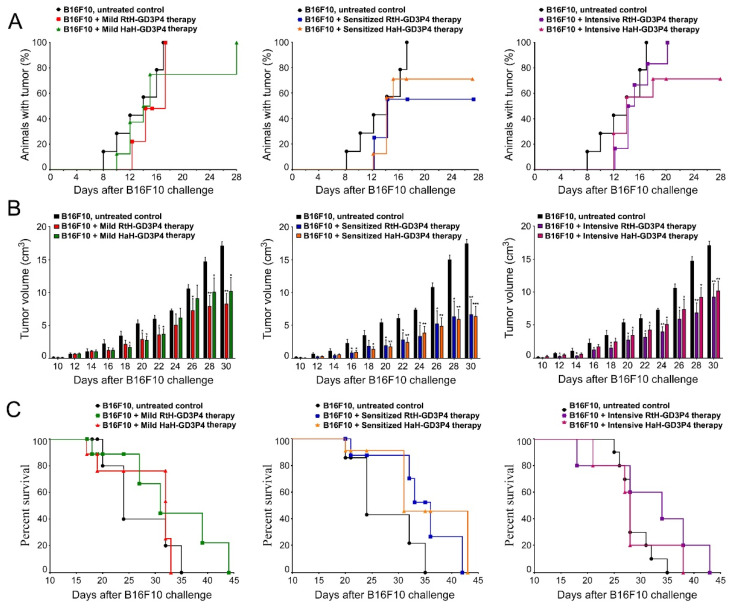
Tumor incidence, tumor growth, and survival analyses in different experimental groups (*n* = 10 mice each) in B16F10 mouse melanoma model. The dynamics of tumor incidence (**A**), tumor growth (**B**), and survival curves (**C**) of experimental B16F10-challenged animals under three different therapeutic approaches with RtH-GD3P4 or HaH-GD3P4 were monitored and compared to controls (untreated B16F10-injected mice). The data are presented as mean ± SD and the *p*-values are calculated using the two-way ANOVA test (* *p* < 0.05; ** *p* < 0.01; *** *p* < 0.001) in comparison to B16F10-bearing mice.

A comparison of the three therapeutic approaches using both anti-tumor vaccines showed a significant suppression of tumor growth rate in the animals treated with either RtH-GD3P4 or HaH-GD3P4 compared to untreated control mice developing tumor (Figure 2B). The strongest delay of tumor formation was found in the animal groups pretreated either with RtH-GD3P4 or HaH-GD3P4, while in the other two therapeutic regimens the administration of RtH-GD3P4 vaccine was more effective within the whole period of observation.

Regarding the tumor incidence, the best results at the end of observation (day 28) were shown in the groups pretreated with the vaccines—70% for HaH-GD3P4 and 60% for RtH-GD3P4, respectively (Figure 2A). A higher tumor incidence was found in the group of mice mildly treated with RtH-GD3P4, but the intensive treatment with the same vaccine postponed the tumor development. The mild treatment with the HaH-GD3P4 delayed the total tumor incidence by two weeks, while in the group of mice intensively treated with the same vaccine, the final score of the tumor incidence was approximately 70%. 

The challenge of the control group of mice with B16F10 cells was lethal, and on day 35 the survival rate was 0%. Even without significant differences, the pretreatment with the two anti-tumor vaccines (RtH-GD3P4 and HaH-GD3P4) prolonged the group survival by 7 days, while under the intensive regimen of therapy the RtH-GD3P4 vaccine showed better survival rate than HaH-GD3P4. The mild administration of HaH-GD3P4 was not effective, with no survivors 33 days later. In contrast, the injection of RtH-GD3P4 resulted in improved survival rates (44 days) compared to the control group ((Figure 2C).

### 2.3. Phenotyping of Tumor Infiltrations

**Figure 3:**
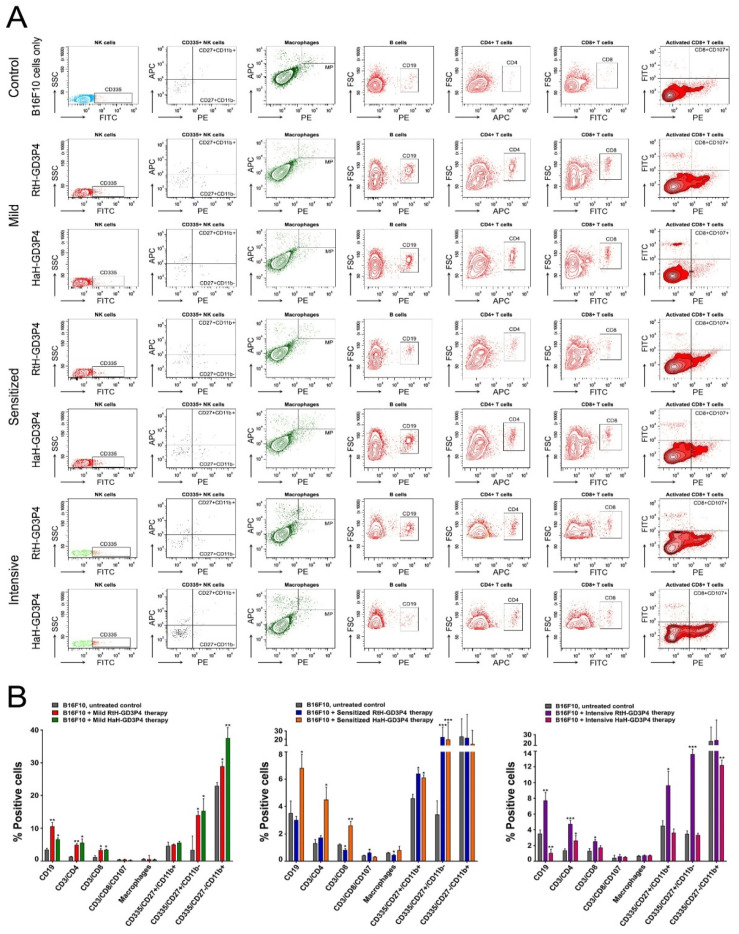
Phenotyping of tumor cell infiltrates. Solid tumors were excised from all experimental animals and the tumor-derived single-cell suspensions were isolated and analyzed with a combination of the following anti-mouse antibodies: CD3e-eFlour450/CD335-FITC/CD11b-APC/CD27-PE; CD11b-APC/F4/80-PE; CD19-PE; CD3e-eFlour450/CD4-APC; CD3e-eFlour450/CD8-FITC/CD107a-PE. Thirty thousand lymphocyte-gated cells from each tube were collected and analyzed by flow cytometry. Representative data of five experiments are shown (**A**). The extracted results from all experiments are presented graphically as the percentage of total viable immune cells (**B**). The data are represented as mean ± SD, *p* values are calculated using the two-way ANOVA test (*n* = 5, * *p* < 0.05; ** *p* < 0.01; *** *p* < 0.001) compared to values from control mice.

Quantitative analysis of the tumor microenvironment was performed by FACS for all B16F10 cells inoculated animals to evaluate the effect of vaccination with RtH-GD3P4 and HaH-GD3P4 using the different approaches of administration. Different tumor-infiltrated lymphocytes were detected following immunization with both vaccines, compared to the untreated tumor-bearing controls (Figure 3). Significant differences were found in the specific cell populations in the sensitized groups pretreated with RtH-GD3P4 or HaH-GD3P4. An increased number of B lymphocytes, CD4+, CD8+ T cells, and NK cells in early maturation stage (CD27+CD11b-) and mature stage (CD27+CD11b+) was found in the group treated with HaH-GD3P4, while higher levels of activated CD8+ and early mature and mature NK cells were observed in mice immunized with RtH-GD3P4 compared to control animals (Figure 3B, middle panel). In contrast, a huge content of CD4, CD8, and CD19 lymphocytes and early mature and mature NK cells was found among the tumor-infiltrated immune cells after immunization of RtH-GD3P4 following the intensive scheme of administration, which correlates with the strong inhibition of the tumor growth (Figure 3B, right panel). An increased number of CD4, CD8 T cells, CD19, and early mature and mature NK cells was found in both groups mildly treated with RtH-GD3P4 or HaH-GD3P4 vaccines, while the percentage of activated CD8 T lymphocytes as well as the macrophages remained unchanged compared to untreated tumor-bearing mice (Figure 3B, left panel).

### 2.4. Tumor-Specific Antibodies Assays

**Figure 4:**
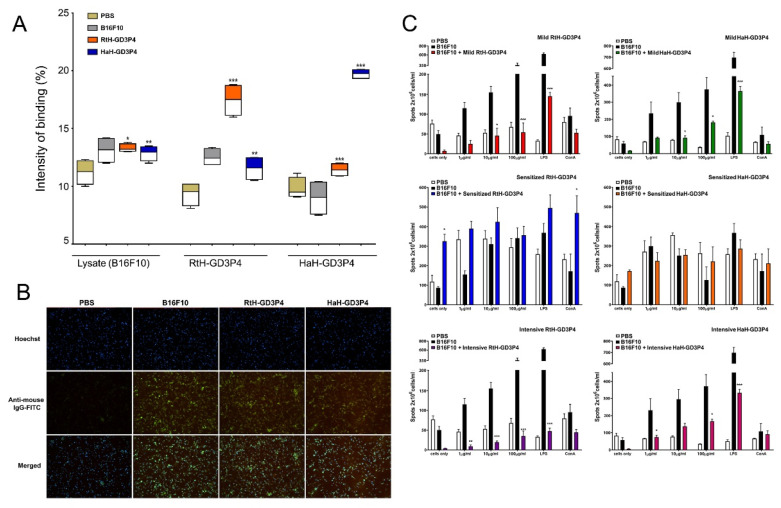
Induction of humoral immune response against B16F10 cells. (**A**) Anti-tumor antibodies in B16F10-challenged animals under the intensive scheme with RtH-GD3P4 or HaH-GD3P4 were measured by Dot blot assay compared to controls. The recognition of B16F10 cell lysate from IgG antibodies is presented as an intensity of binding (%) using Image J software. (**B**) Indirect immunofluorescence analysis. The fixed B16F10 cells were incubated with mouse sera from sensitized therapy-treated animal groups (dilution 1:25) followed by anti-mouse IgG-FITC antibody and Hoechst 33342 staining. Magnification 20×. (**C**) ELISpot assay for evaluation of vaccines’ therapy. Isolated mouse splenocytes from all experimental animals were exploited for counting of anti-B16F10 IgG antibody-secreting plasma cells. The number of spots in the test-wells corresponding to B16F10-specific plasma cells was compared to untreated B16F10-bearing controls. All samples (**A**,**C**) were triplicated, and mean ± SD values were presented for each group; *p* values were calculated using the two-way ANOVA test. (* *p* < 0.05; ** *p* < 0.01; *** *p* < 0.001). A representative of three independent experiments is shown.

We tested individually the collected sera from untreated disease-free mice, PBS-treated B16F10 tumor-bearing animals, and from mice immunized with RtH-GD3P4 or HaH-GD3P4 under the intensive scheme by Dot blot assay for induction of tumor-specific antibodies after vaccine therapy. The antibodies in sera from the B16F10 tumor-bearing group and vaccine-treated groups recognize equally the B16F10 cell lysate loaded on the membranes, compared to the healthy controls (Figure 4A). The presence of high levels of antibodies recognizing loaded RtH-GD3P4 or HaH-GD3P4 in the groups immunized with the respective vaccines is not surprising, demonstrating the share of specific anti-tumor epitope antibodies in all inoculated animals with B16F10 cells plus extra anti-Hcs antibodies in the specifically treated groups.

The affinity of the generated anti-tumor antibodies to intact B16F10 cells was visualized by fluorescent microscope analysis. The high expression of GD3 on melanoma cell surface was used for fluorescent assay using the same sera from Sense-treated animal groups. It seems that the mice challenged with B16F10 cells generate similar and even higher levels of tumor-recognizing antibodies such as those of the tumor-bearing groups immunized with RtH-GD3P4 or HaH-GD3P4 vaccines under the sensitized therapy compared to untreated intact mice (Figure 4B).

### 2.5. The Vaccine Therapy Did Not Rise the Th2 Immune Response

The induction of anti-B16F10 IgG antibody-producing plasma cells after RtH-GD3P4 or HaH-GD3P4 administration was assessed by ELISpot assay using spleen cells from all treated in vivo tumor-bearing mice. Generally, the effect of the vaccine therapy on the number of tumor-specific antibody-secreting plasma cells under different regimens of immunization was not stronger than the measured tumor-induced Th2 response itself without therapy (Figure 4C). Immunization with either RtH-GD3P4 or HaH-GD3P4 under mild and Intensive schedule as well as in the group of mice pretreated with HaH-GD3P4 (Sensitized HaH-GD3P4 group) did not result in generation of high levels of anti-B16F10 IgG antibody-producing plasma cells and did not even reach the number of plasma cells in tumor-bearing mice without treatment. An increase in the number of plasmocytes secreting antibodies with the same specificity was found in the Sensitized RtH-GD3P4 group. A higher significant value was measured without ex vivo stimulation with RtH-GD3P4 compared to untreated tumor-bearing mice.

### 2.6. Cytokine Measures

**Figure 5:**
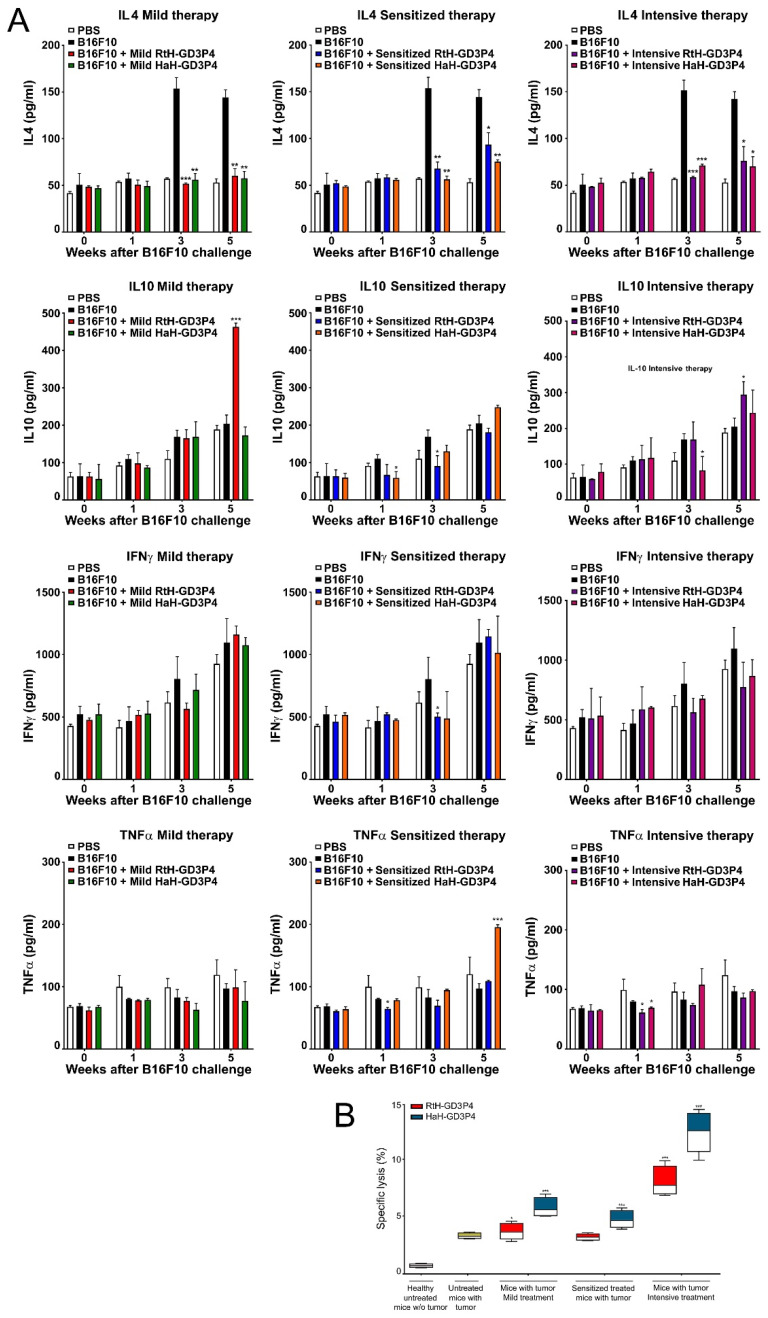
Cytokines production and CTL generation in the animals after in vivo RtH-GD3P4 or HaH-GD3P4 administration. (**A**) Serum levels of IL4, IL10, TNFα, and IFNγ were measured by ELISA. (**B**) Resulted CTL activity of mouse splenocytes after the therapies. The spleen cells from all vaccine-treated animals and control group were isolated at the 25th day after B16F10 cells challenge and were co-cultured as effector cells at a ratio 40:1 with the target B16F10 cells. The released LDH in the supernatants were measured by commercial CytoTox assay and the values were calculated. All samples were triplicated and the data are presented as mean ± SD for each group; *p* values were calculated using the two-way ANOVA test (* *p* < 0.05; ** *p* < 0.01; *** *p* < 0.001) in comparison to B16F10-bearing mice. Representative data of four independent experiments are shown.

The serum levels of IL4, IL10, IFNγ, and TNFα were measured weekly in C56B6 mice sera from all experimental groups using quantitative ELISA (Figure 5A). The treatment with both vaccines under all regimens of therapy resulted in significantly lower IL4 production in all measured points compared to control tumor-bearing group. The same therapy did not lead to significant differences in IFNγ values between the test groups. The mice under the mild and intensive regimen of treatment with RtH-GD3P4 produced significantly higher serum levels of IL10 at the end point of observation (5th week) compared to the control and HaH-GD3P4 treated groups. It was not observed for the sensitized therapy RtH-GD3P4 group, but weak increase of the same cytokine was measured in the sensitized therapy HaH-GD3P4 group at the end point of observation. 

The serum values of TNFα in the tested groups also did not exhibit a stable tendency of any differences between the regimens of treatment, with some points of significance only.

### 2.7. Generation of CTLs

We exploited a CTL activity test to determine the generated B16F10-specific cytotoxic T cells after vaccines’ therapy under different regimens. Isolated spleen cells from all terminal mice were examined against melanoma tumor cells, and the released LDH after lysis was measured. The significant highest cytotoxic values were found in the groups intensively treated with both vaccines, but HaH-GD3P4-vaccinated animals exhibited better results than the RtH-GD3P4-immunized group. 

The other vaccinations (mild or sensitized scheme) with HaH-GD3P4 showed a moderate cytotoxic activity in the treated mice, while the treatment with RtH-GD3P4 under the same regimens did not induce high values of cytotoxic T lymphocytes compared to untreated tumor-bearing controls (Figure 5B). The weak increase of CTL was determined in the mildly treated group with RtH-GD3P4.

### 2.8. Tumor M1/M2 Phenotyping

**Figure 6:**
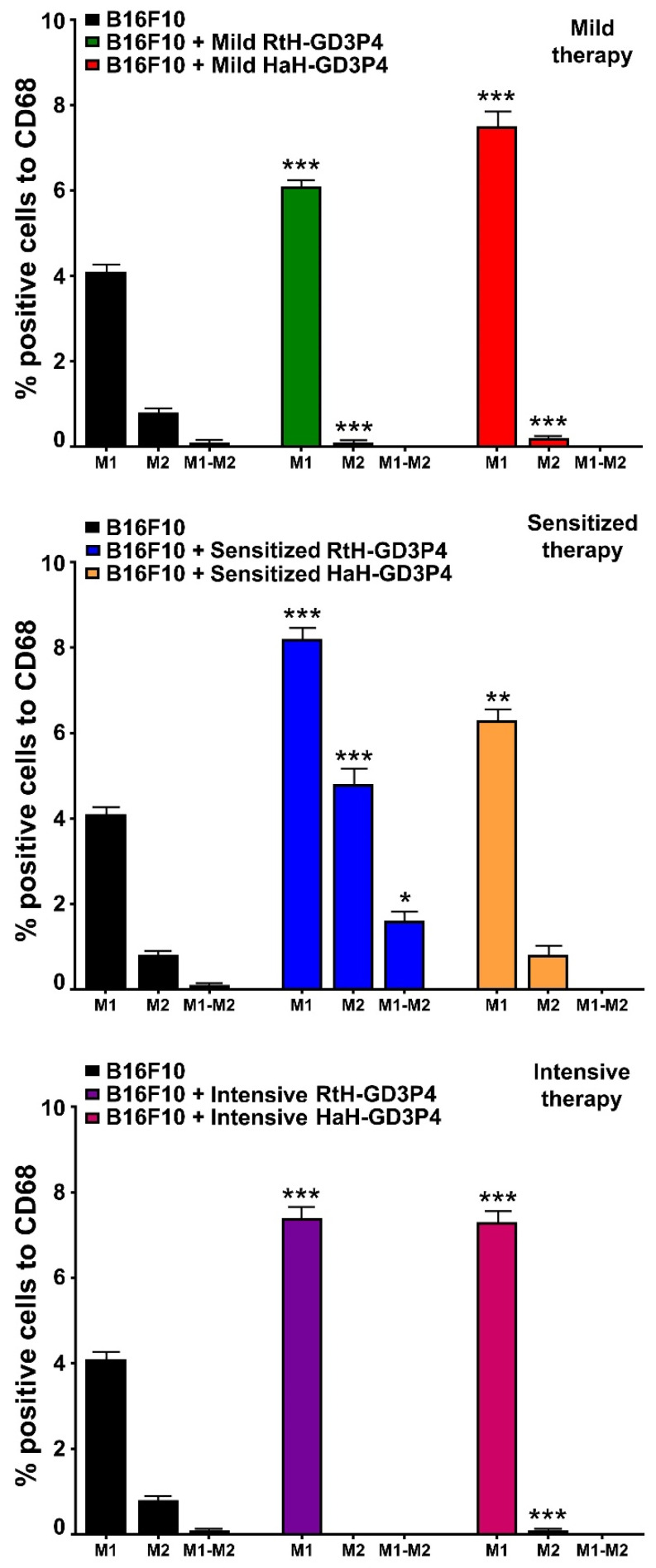
M1/M2 discrimination in tumor-infiltrating macrophages. The isolated tumor-derived single-cell suspensions from B16F10-challenged mice and treated under three different therapeutic approaches with RtH-GD3P4 or HaH-GD3P4 were analyzed with combination of the following macrophage markers: CD68-PE/CD86-APC or CD68-PE/CD163-Brilliant Violet 421 antibodies. Thirty thousand CD68-positive cells were analyzed from each sample for M1/M2 monitoring using flow cytometry. The extracted results from all experiments are presented graphically as the percentage of total CD68-positive cells. The data are represented as mean ± SD, *p* values are calculated using the two-way ANOVA test (*n* = 5, * *p* < 0.05; ** *p* < 0.01; *** *p* < 0.001) compared to values from control mice.

The administration of RtH-GD3P4 or HaH-GD3P4 to experimental animals generates significantly higher levels of tumor-infiltrated M1 macrophages, compared to tumor-bearing control mice (Figure 6). Low numbers of M2 macrophages in the solid tumors were counted in both vaccine-treated groups under mild and intensive schemes of immunization, while elevated levels were detected in both sensitized therapy-treated groups. An increased level of double positive (CD86+/CD163+) M1-M2 macrophages was found in the RtH-GD3P4 sensitized therapy treated group.

No significant differences for M1/M2 ratio in the spleens were found between the groups (data not shown).

## 3. Discussion

Numerous natural products and their compounds have been investigated for anticancer therapy. These new molecules are an important source for treatment and clinical trials looking for effective mechanisms to inhibit tumor growth. Therefore, increased attention has been paid to the anti-cancer potential of agents, isolated from marine organisms.

The intact immune system is recognized as the critical factor in the anti-tumor resistance associated with both the onset of cancer and its treatment. The intensive efforts for tumor therapy are directed to enhance the tumor-specific immune response in several ways. Most immune-related approaches are based on T-cell-directed therapy such as checkpoint blockade, anti-cancer vaccines, adoptive dendritic and T cell transfer, and CAR-T administration [26].

Hcs isolated from arthropods and mollusks are widely used for anti-cancer therapy alone or as protein-carriers for TACAs [13,14,15,16,22,27]. These xenogenic proteins exhibit multi-face activating properties toward innate and adaptive immune response and trigger Th1 polarization. It is due to interaction of the unique glycosylation patterns of Hcs with C-type lectin receptors such as mannose receptor (MR), and toll-like receptor (TLR) 4 both on dendritic cells (DCs) and on macrophages [28,29,30]. These activated antigen-presenting cells (APCs) trigger the production of pro-inflammatory cytokines and signal internalization to either T and B lymphocytes engaging Hc-specific cellular and humoral immune responses. The exhibited Hcs’ properties promote the anti-tumor experiments in different mouse cancer models, which is a base for the development of many therapeutic anti-cancer vaccines.

Neoantigen cancer vaccines require precise selection of epitopes for the formulation of desired targets, but the short synthetic peptides/proteins are weakly immunogenic. Improved delivery of epitopes to dendritic cells and macrophages with subsequent processing to immune cells is essential for the success or failure of vaccines [26]. Identification of tumor-associated antigens (TAAs) is a necessary step for a potential target definition and design of cancer vaccines [6,31,32]. In the last 20 years, a variety of TAA-based vaccines have been developed in pre-clinical and clinical trials, exploring different delivery methods with the aim to improve the immune response: adjuvants, nanoparticles, receptor-targeted complexes, and protein-carriers. Among them, numerous Hcs have been widely used alone or as protein-carriers for the development of cancer vaccines [14,15,20,21]. The high content and variety of carbohydrates and oligosaccharides (such as mannose) expression found in the Hcs are a prerequisite for sharing of epitopes with TAAs resulting in different anti-cancer effects among them [10,15,28,30,33].

The main difference between tumor and normal cells is the expression ratio and the type of carbohydrates on their cell surfaces marked as tumor-associated carbohydrate antigens (TACAs) conditioned in two main groups: glycoprotein antigens (TACAs are bound to proteins), widespread in epithelial cancers, and glycolipid antigens (TACAs are bound to ceramides) shared by melanoma, lung, ovarian, breast, colon, and prostate cancers [24]. Gangliosides (GD2, GD3, GM2, GM3, and fucosyl-GM1) are classified as a group of glycolipids which consists of sialic acid, carbohydrates, and ceramides. Normally, they participate in numerous receptor-mediated signaling pathways of intact cells, but overexpression of these gangliosides is found on the outer leaflet of malignant cells. 

Several novel TACA-based cancer vaccines have been developed during the last decades related to the new technologies for TACAs synthesis. The main limitation of these vaccines for clinical expansion is the fact that TACAs are T-cell-independent antigens and induce a weak humoral immune response [34]. Covalent binding of carbohydrates to T cell-dependent protein-carriers such as KLH, BSA, ovalbumin, or toxoids could enhance the complex immunogenicity and turn the immune response to the T-arm of the immunity. As main protein-carriers, Hcs are widely used for TACA-based cancer vaccine constructions in several tumor models and clinical trials: Globo-H-KLH conjugate vaccine for breast cancer [35]; Globo-H-GM2-sTn-TF-Tn-KLH conjugate vaccine for ovarian cancer [36]; and GM2-KLH conjugate for melanoma treatment [37].

Melanocytes-derived skin cancer expressed elevated levels of the gangliosides GM3, GD3, GM2, GD2, which could be found in the melanoma tumor microenvironments [36]. The administration of conjugate GM2-KLH/QS-21 to melanoma patients in clinical trials indicated promising results and elicits anti-GM2 immune response [38]. Another two conjugates of GD2 mimotopes to Hcs CCH and FLH have been used successfully in the B16F10 murine model of melanoma [14]. Here, the overexpression of GD2 has been used as a target for antibody-dependent cell-mediated cytotoxicity (ADCC) and complement-dependent cytotoxicity promoted by the post-immunization generated anti-GD2 antibodies [14]. In the present study, we used the same classical C57B6 mouse model challenged with B16F10 melanoma cell line. GD3 is another possible target in melanoma, and high expression levels of this ganglioside are displayed on the surface of the malignant cells in contrast to the very low expression in normal cells and tissues [25]. Instead of intact form of GD3, we used GD3-mimotope peptide Ac-RHAYRSMAEWGFLYS-Ahx-K-CONH2 (GD3P4) for conjugation to both Hcs RtH and HaH in order to vaccinate tumor-bearing C57B6 mice. The engineered constructs RtH-GD3P4 and HaH-GD3P4 have been administered in vivo under three different regimens of treatment in the B16F10 mouse melanoma model. Summarizing the results obtained, we can conclude that both vaccine constructs suppressed the tumor size growth, prolonged the survival of treated mice, and affected the tumor incidence. The highest capacity to inhibit tumor growth, survival, and tumor incidence was exhibited by both vaccines under the regimen with pretreatment, while the mild therapy of the animal groups provides only a weak benefit. In the same way, the groups of mice treated under intensive regimen also showed lower tumor growth rate and improved survival compared to the control tumor-bearing mice. Palacios et al. have also shown promising results using GD2 mimetic peptide coupled to CCH and FLH under the regimen with pretreatment in the same model [14].

Several authors reported the way the enhanced antitumor antibody formation prolonged the survival in some cancers [39]. Recently, we have shown that the Hcs induced high titers of antibodies, which cross-reacted with tumor cells in the C-26 murine model of colorectal carcinoma [20,21]. It was not surprising that Hcs conjugated to GD3P4 generated high titers of IgG antibodies, which recognize the construct itself but also recognize lysate from B16F10 tumor cells, confirmed by indirect immunofluorescence assay and Dot blot. Surprisingly, high titers of antibodies against B16F10 cells were also generated in the control tumor-bearing mice without any anti-tumor mechanism involvement. Furthermore, the ELISpot assay for B16F10-specific antibody-producing plasma cells showed the highest number of spots in untreated tumor-bearing mice compared to animals treated with both vaccines under all regimens of therapy. The only exception is the group of mice pretreated with RtH-GD3P4, which elicited a significantly higher number of plasma cells producing IgG antibodies with the same specificity. It seems that without activation of cytotoxic CD8 and NK cells, the anti-tumor antibodies alone are not able to build tumor-suppressive immune response. 

Analysis and evaluation of tumor-infiltrated cells provide important information for precise mechanisms of anti-tumor therapy. Generally, we found a changed ratio of immune cell quantity and activity, leading to varying degrees of success depending on the vaccine and regimen of treatment. High levels of CD4+ and CD8+ T cells in all groups vaccinated with RtH-GD3P4 or HaH-GD3P4, compared to controls, are a potency factor for effective anti-tumor immune response. RtH-GD3P4 exhibited better properties as inducer of early mature and mature NK cells in all schemes of immunization as well as partially for activated CD8+ T lymphocytes in the sensitized group, while HaH-GD3P4 induced elevated levels of early mature and mature NK cells in mild and sensitized treated groups.

Tumor-infiltrated B cells could secrete anti-B16F10 antibodies, adding an extra beneficial effect to CD8+ and NK cells infiltration and implementing antibody-dependent cell cytotoxicity (ADCC). In contrast to the lower serum values of anti-B16F10 antibodies and the low number of antibody-producing plasma cell with the same specificity in the spleens of vaccine-treated groups, an increased number of tumor-infiltrated B lymphocytes were counted in the animals from RtH-GD3P4 mild and intensively treated groups and in the mice from HaH-GD3P4 mild and sensitized treated groups. Altogether, these results generally correspond to the values of CTL in the animal groups, provided that CTL was performed with splenocytes but not with tumor-infiltrated lymphocytes. Thus, probably together with the missing tumor-induced regulatory T cells could explain the highest values of CTL, obtained in the intensively treated with HaH-GD3P4 mice, which was not supported by high CD8+ and NK levels. Subsequent precise T cell subtypes analysis may explain in detail the role of each cell sub-population in generating anti-tumor immune responses. 

Macrophages are key participants in tumor pathogenesis. They can be detached into two general classes (M1 and M2) based on their function and are regulated by the tumor microenvironment. M1 polarized macrophages are activated by TNFα and IFNγ and promote pro-inflammatory activity, which supports the cytotoxic potential and possesses anti-tumor functions [40,41]. M2 tumor-associated macrophages (TAMs) are stimulated by anti-inflammatory cytokines (IL4 and IL13) and enhance the tumor growth by down-regulation of the inflammatory response. Their responsiveness to TLRs and IFNγ stimulation is also decreased. It was reported that the mannose-containing carbohydrates of KLH, CCH, and FLH interact with human MR in glycan-dependent manner on the surface of APCs and together with TLR4 induce M1 phenotype macrophage profile with enhanced antitumor activity [28]. The same treatment downregulated M2 cytokines production.

The vaccination of mice with RtH-GD3P4 or HaH-GD3P4 did not contribute to enhanced macrophages infiltration, compared to untreated tumor-bearing animals. The subsequent analysis of M1/M2 ratio shows significant M1 increase in all vaccine-treated groups, compared to control mice. Hcs activation of M1 macrophages via TLR4 leads to enhanced surface expression of CD40, CD80, CD86, MHC-I, and MHC-II molecules and TNFα production involved in cell maturation and activation of NK cells [27,29,41]. In our study, high TNFα production was measured in mouse sera in the end-point of sensitized treatment with HaH-GD3P4 only, while no significant differences for IFNγ production were found between the groups. It seems that the local intra-tumor IFNγ production by tumor-infiltrated NK, CD4 Th1, and CD8 cytotoxic T lymphocytes together with TNFα supported the generation of high levels of specific antitumor CTLs leading to tumor growth suppression. 

As expected, elevated levels of IL4 have been found in the sera of tumor-bearing mice produced by tumor-infiltrating lymphocytes and supporting activation of NF-κB in M2 macrophage phenotype. Similar IL4 increase and M2 prevalence were found in the tumor microenvironment of cancer patients [42].

## 4. Materials and Methods

### 4.1. Antibodies

Fluorescein isothiocyanate (FITC)-conjugated anti-mouse CD8 and CD335, eFlour450 –conjugated anti-mouse CD45 and CD3; Phycoerythrin (PE)-conjugated anti-mouse F4/80, CD107a and CD27; Allophycocyanin (APC)-conjugated anti-mouse CD4 and CD11b and Pacific Blue-conjugated anti-mouse CD19 mAbs (eBioscience, Frankfurt, Germany) were used for fluorescence-activated cell sorting (FACS) experiments. Alkaline phosphatase (AP)-conjugated anti-mouse IgG (Sigma-Aldrich, Taufkirchen, Germany) antibody was used for Enzyme-linked Immunospot assay (ELISpot) and Dot blot analysis.

### 4.2. Cell Line

Murine melanoma cell line B16F10 (ATCC^®^ CRL6475 ™) was kindly provided by Dr. Sergej Tomic, Institute for the Application of Nuclear Energy (INEP), University of Belgrade and was cultured in complete Dulbecco’s Modified Eagle Medium (DMEM, Gibco, Gaithersburg, MD, USA) supplemented with 10% heat-inactivated fetal calf serum (FCS), 1 mM sodium pyruvate, 4 mM L-glutamine and antibiotics at 37 °C/5% CO_2_. The 80% confluent cell monolayer was treated by accutase solution (eBioscience) and monocellular suspension was prepared by cell strainers (BD Biosciences, Mountain View, CA, USA).

### 4.3. B16F10 Cell Lysate Preparation

B16F10 cells were cultured in complete DMEM medium, and monocellular suspension was prepared and centrifuged. The pellet was collected, suspended in PBS (1 × 10^7^ cells/mL), and proceeded to 7-fold cycles of freeze-thaw (from −80 °C to 37 °C). The cell suspension was centrifuged, and the supernatant was collected. The total protein content was measured spectrophotometrically at λ = 280 nm.

### 4.4. Mice

Female 6-week-old C57BL/6 mice were obtained from The Jackson Laboratory (Bar Harbor, ME, USA). The mice were kept under specific pathogen free (SPF) conditions at the Institute of Microbiology, Bulgarian Academy of Sciences and housed at 22–24 °C with a light/dark cycle of 12/12 hrs. All animal experiments were carried out in strict accordance with the Guidelines for the Care and Use of Laboratory Animals of the European Union (EU Directive 2010/63/EU), and the manipulations were approved by the Animal Care Commission at the Institute of Microbiology (N286/16.04.2021) in accordance with the national regulations.

### 4.5. Isolation and Purification of RtH and HaH

RtH and HaH were isolated from the hemolymph of marine gastropod *Rapana thomasiana* or of terrestrial snails *Helix aspersa,* respectively, as described [43,44,45]. After subsequent purification by gel filtration chromatography and additional endotoxin removing by pass through Detoxi-Gel column (Detoxi-Gel column, Thermo Fisher Scientific, Rockford, IL, USA), both concentrated Hcs were tested for residual endotoxins under pyrogen-free conditions using Limilus Amebocyte Lysate coatest gel (LAL) (Chromogenix AB, Molndal, Sweden).

### 4.6. Construction of Vaccine Molecules Containing GD3-Mimicking Peptide and Hcs

The GD3-mimotope peptide (RHAYRSMAEWGFLYS) of the tumor-associated ganglioside GD3 was found by phage display with the GD3-specific monoclonal antibody 4F6 [46]. The peptide Ac-RHAYRSMAEWGFLYS-Ahx-K-CONH_2_ (GD3P4) was synthesized with >96% purity from Caslo Laboratory (Lyngby, Denmark). 

The vaccine molecules were constructed by chemical conjugation of GD3P4 peptides to RtH or to HaH using the zero-length crosslinking agent EDC (1-ethyl-3-(3′-dimethylaminopropyl) carbodiimide.HCl), (Sigma-Aldrich) [47]. During the peptide synthesis, an Ahx-K-NH_2_ linker carrying free ε-amino groups of lysine residues was introduced to the C-end of the peptides in order to bind the Hcs molecules. The Hcs (0.1 mg/mL in 0.1 M phosphate buffer, pH 6.0) were mixed with GD3P4 peptides dissolved in N, N-dimethylformamide (Sigma-Aldrich)/PBS in 200-fold molar excess with respect to the Hcs. For the initiation of the protein conjugation, EDC was added in 600-fold molar excess to the Hcs to couple the available free carboxyl groups from the Hcs to the ε-amino groups of lysine residues. The reaction mixture was left overnight at 4 °C upon stirring, then dialyzed against PBS to remove the excess of reagents. The final engineered vaccine molecules RtH-GD3P4 and HaH-GD3P4 were concentrated by ultrafiltration through a filter XM100 (Amicon, Darmstadt, Germany) and the protein concentrations were measured by spectrophotometer at a wavelength of 280 nm.

### 4.7. Mouse Model of Melanoma and Treatment Schedule

Single cell suspension from B16F10 murine melanoma cell line was prepared as described above. Seven groups of female 10-week-old C57BL/6 mice (10 animals per group) were randomized and challenged subcutaneously (*s.c.*) into the right flank with B16F10 cells (1.5 × 10^5^ cells/mouse). After palpable solid tumor formation, two groups of animals were immunized every week intratumorally with 100 μg/mouse RtH-GD3P4 (Mild RtH-GD3P4 group) or with HaH-GD3P4 (Mild HaH-GD3P4 group) for 4 weeks.

Another two groups of mice were treated intensively with 100 μg/mouse of RtH-GD3P4 or HaH-GD3P4 once a day starting on the next day after the B16F10 cells challenge by *s.c.* immunization in the area of tumor cells inoculation for 7 consecutive days (Intensive RtH-GD3P4 and Intensive HaH-GD3P4 groups), followed by intratumoral injection of the same amounts of RtH-GD3P4 or HaH-GD3P4 once a week for 4 weeks.

Another two groups of animals were pretreated with 100 μg/mouse of RtH-GD3P4 or HaH-GD3P4 14 days, prior to the challenge with B16F10 cells (Sensitized RtH-GD3P4 and Sensitized HaH-GD3P4 groups). Furthermore, the mice were injected once a week with the same amounts of RtH-GD3P4 or HaH-GD3P4 by *s.c.* immunization in the area of tumor cells inoculation for 4 weeks, starting on the next day after the B16F10 cells challenge.

Two control groups of mice were immunized with RtH-GD3P4 or HaH-GD3P4 under the scheme of sensitized treatment without B16F10 cells challenge. One more control group was injected with PBS only also without B16F10 cells challenge. Another control group of mice was challenged with B16F10 cells and injected with PBS only. Every week the mice from all groups were bled, and the sera were stored frozen at −70 °C.

### 4.8. Tumor Assessment

Tumor incidence and growth were estimated every two days by measuring the solid tumor width and length using a manual caliper by the same examiner to minimize bias. After day 30, the number of survivors in the groups was not sufficient for statistical significance. Tumor volumes were calculated using the formula:

Volume (cm^3^) = width^2^ × length × 0.52 (1)

The mice from all groups were observed for 6 weeks and the survival rates of the test groups were compared with the survival of the untreated control group.

### 4.9. Phenotyping of Tumor Infiltration by Flow Cytometry

The analysis of intra-tumor cell infiltration phenotype was performed by flow cytometry. Solid tumors from all terminal animals in vaccine-treated and control groups were isolated at the 25th day after B16F10 cells challenge, and the single-cell suspensions were prepared by grinding them through a cell strainer. The erythrocytes were removed by incubation with lysis buffer (0.16M NH_4_Cl, 0.01M KHCO_3_, 0.12mM EDTA, pH 7.0) for 2 min., and the cells were washed twice with FACS buffer (PBS containing 2.5% FCS and 0.05% sodium azide) and then counted. The cells were transferred into FACS tubes (BD Falcon, BD Biosciences, San Diego, CA, USA) (2 × 10^5^ cells/tube) and incubated for 30 min on ice with one of the following mixes of anti-mouse antibodies: eFlour 450-conjugated CD3e antibody, FITC-conjugated CD335 antibody, APC-conjugated CD11b antibody and PE-conjugated CD27 antibody for NK population; APC-conjugated CD11b antibody and PE-conjugated F4/80 antibody for Macrophage population; PE-conjugated CD19 antibody for B cell population; eFlour 450-conjugated CD3e antibody and APC-conjugated CD4 antibody; FITC-conjugated CD8 antibody and PE-conjugated CD107a antibody for CD4 and CD8 T cell populations. Thirty thousand lymphocyte-gated cells from each tube were collected and analyzed with a BD LSR II flow cytometer using the Diva 6.1.1. software (BD Biosciences, Mountain View, CA, USA).

### 4.10. Dot-Blot Analysis for the Presence of Tumor-Specific Antibodies

Dot-blot analysis was performed for detection of tumor-specific antibodies in mouse sera from disease-free, B16F10 tumor-bearing, and from mice immunized with RtH-GD3P4 or HaH-GD3P4 following the intensive scheme. Ninety-six-well ELISpot plates (Millipore, Bedford, MA, USA) were activated for 1 min with 35% ethanol and washed with PBS (pH 7.4). B16F10 cell lysate, or RtH-GD3P4, or HaH-GD3P4 were loaded (150 μg/mL in PBS) into individual wells of plates for 16 h at 4 °C. Later, the plates were washed three times with TBS/0.05% Tween 20 (T-TBS, pH 7.4) and blocked for 30 min at room temperature with a 1% bovine serum albumin (BSA, Sigma-Aldrich) in T-TBS. After washing, the plates were incubated with diluted murine sera (20×) for 1 h, followed by incubation with an AP-conjugated anti-mouse IgG antibody for 1 extra hour. The membranes were developed by NBT/BCIP (5-bromo-4-chloro-3-indolyl-phosphate/nitro blue tetrazolium) substrate (Sigma) and measured by C.T.L Immunospot S5 Versa Analyzer (Bonn, Germany). The Dot-blots were analyzed using Image J software and the data were processed by GraphPad Prism 5.0 software (GraphPad Software, Inc., San Diego, CA, USA).

### 4.11. Immunofluorescence Assay for Detection of Anti-B16F10 Cell Antibodies

Murine melanoma cell line B16F10 (2 × 10^6^ cells) was cultured in 6-well plates (TPP, Trasadingen, Switzerland) on sterile cover slips in supplemented DMEM for 24 h at 37 °C/5% CO_2_. Then, the cells were fixed with 4% paraformaldehyde in PBS for 4 min. at room temperature and then washed 2 times with 0.05% Tween 20–PBS for 3 min. each, followed by washing with PBS. The unspecific binding sites were blocked with 2% BSA in PBS for one hour and subsequently the cells were incubated with mouse sera from Sense-treated animal groups for 30 min. at room temperature (dilution 1:25) and washed again. The plate was incubated with rabbit anti-mouse IgG-FITC antibody (Sigma-Aldrich, F9137) for 1h at room temperature and was washed again. Next, Hoechst 33342 Staining Dye Solution (abcam, ab228551, dilution 1:8000) was added for 10 min. and after washing the cells were covered with Fluoromount™ Aqueous Mounting Medium (Sigma-Aldrich, cat № F4680). The samples were analyzed by a fluorescent microscope (Leica DM6B, Wetzlar, Germany) and the resulting images were processed with Leica Application Suit X 3.7.4.23463.

### 4.12. ELISpot Assay for Specific Anti-B16F10 IgG Antibody-Secreting Cells

The effects of vaccine therapy on the number of IgG anti-B16F10 antibody-producing plasma cells were assessed by ELISpot as described (24). Briefly, the experimental animals were sacrificed on the 25^th^ day after the B16F10 cells challenge. Splenocytes from all mice treated in vivo with PBS only, B16F10 tumor-bearing, and from animals challenged with B16F10 and immunized with RtH-GD3P4 or HaH-GD3P4 following the different schemes (see the Treatment schedule section) were isolated and further cultured (2 × 10^6^/mL) ex vivo for 3 days at 37 °C/5% CO_2_ in the presence of different concentrations (2, 20, or 200 µg/mL) of respective vaccines in supplemented DMEM medium. 

Next, B16F10 lysate-coated (150 µg/mL) 96-well ELISpot plates were blocked with 1% gelatin and incubated with the previously pre-cultured cells for additional 4 h at 37 °C/5% CO_2_. Later, the membranes were developed as described under the dot-blot section and the resulting spots corresponding to anti-B16F10 antibody-producing plasma cells were counted and analyzed by C.T.L Immunospot S5 Versa Analyzer.

### 4.13. Cytokine Detection

Tumor necrosis factor alpha (TNFα), Interferon gamma (IFNγ), IL4, and IL10 levels were measured in mouse sera using ELISA sets (Abcam, Cambridge, UK) according to the manufacturer’s instructions.

### 4.14. Cytotoxicity Assay

Generated cytotoxic T lymphocytes specific for tumor B16F10 cells were determined by a non-radioactive cytotoxicity assay. B16F10 murine melanoma cell line was cultured as described above, and upon reaching confluence the cells were washed, detached by accutase solution, and transferred to a 96-well culture plate (1 × 10^4^ cells /well). Splenocytes were isolated from all experimental animals, sacrificed at the 25th day after B16F10 cells challenge (see ELISpot section) and have been used as effector cells in a cytotoxic assay at a target (B16F10 cells) to effector cells ratio 1:40. The effector spleen cells were added (4 × 10^5^ cells/well) to the target cells in the experimental wells, and the specific lysis (%) was determined by the CytoTox (Promega, Madison, WI, USA) kit according to the instructions.

### 4.15. Phenotyping of Tumor M1 and M2 by Flow Cytometry

In order to determine the phenotype of tumor-infiltrated macrophages, we used flow cytometry. Solid tumors and spleens from all terminal animals in vaccine-treated and control groups were isolated, and the cell suspensions were prepared as described above. The cells were washed with FACS buffer and incubated with following mixes of anti-mouse antibodies for 20 min. at 4 °C: CD68-PE/CD86-APC antibodies, CD68-PE/CD163-Brilliant Violet 421 antibodies. Thirty thousand CD68-positive cells were analyzed from each sample to discriminate M1 and M2 macrophages with a BD LSR II flow cytometer using the Diva 6.1.1. software (BD Biosciences).

### 4.16. Statistical Analysis

All statistical analyses including survival significance were performed with Prism software from GraphPad (San Diego, CA, USA). The two-way ANOVA test was used to determine differences between each two groups, and values in the figures correspond to mean ± SD. All Dot blot, Cytokine, ELISpot, and cytotoxicity samples were triplicated. A value of *p* < 0.05 was considered as statistically significant. Survival significance was determined using the method of Kaplan and Meier.

## 5. Conclusions

The protein-engineered vaccines RtH-GD3P4 and HaH-GD3P4 exhibited a strong anti-tumor immune response in the B16F10 murine melanoma model. The obtained results showed several mechanisms for tumor suppression after immunization with two Hc-based epitope vaccines under different regimens of treatment. This study demonstrated a promising approach for cancer therapy having potential applications for cancer vaccine research.

## Data Availability

Not applicable.
